# Switching behaviour of dSTORM dyes in glycerol-containing buffer

**DOI:** 10.1038/s41598-020-70335-0

**Published:** 2020-08-13

**Authors:** Nora C. Goossen-Schmidt, Marco Schnieder, Jana Hüve, Jürgen Klingauf

**Affiliations:** 1 Fluorescence Microscopy Facility Münster (FM)², Institute of Medical Physics and Biophysics, Center For NanoTechnology (CeNTech), Heisenbergstraße 11, 48149 Münster, Germany; 2 Department of Cellular Biophysics, Institute of Medical Physics and Biophysics, University of Münster, Robert-Koch-Straße 31, 48149 Münster, Germany

**Keywords:** Single-molecule biophysics, Super-resolution microscopy

## Abstract

To suppress optical aberrations caused by refractive index mismatch, we employ glycerol-immersion objectives in conjunction with fused silica cover glasses and imaging buffers with a high glycerol content. Here we demonstrate that the addition of glycerol to the buffer does not degrade the switching behaviour of the dyes Alexa Fluor 647 and Alexa Fluor 568 in dSTORM measurements, which shows that this approach is suitable for dSTORM. Additionally, we report evidence that sealed sample geometries as used in our experiments reduce photobleaching due to the lower influx of oxygen into the imaging buffer.

## Introduction

Single molecule localisation microscopy (SMLM) elegantly circumvents the diffraction limit in fluorescence microscopy by determining the spatial coordinates of single dye molecules, which can be done with a precision more than one order of magnitude better than the optical resolution^1–3^ if the density of fluorescing molecules is low enough to avoid overlapping signals. This is achieved by employing photo-switchable or photo-activatable dyes. During imaging, most of the dye molecules are in a non-fluorescent state, from which they switch stochastically into the fluorescent state with a sufficiently low probability, until they reversibly switch back into the non-fluorescent state or irreversibly bleach. Consequently, the switching properties of a dye greatly influence the resulting image quality^4^.

In direct stochastic optical reconstruction microscopy (dSTORM), a variant of SMLM which employs conventional organic dyes, switching is mediated via reducing agents that reduce dye molecules in the triplet state to a non-fluorescent radical anion state. By reacting with oxygen, the molecule can return to the ground state from which it may again be excited to fluoresce^5,6^. The oxygen concentration of the buffer needs to be kept low to prevent photobleaching and too high reactivation rates. Therefore, an oxygen scavenging system is usually added to the buffer, consisting of glucose, glucose oxidase and catalase.

Since after the acquisition process, the molecules’ coordinates are usually obtained by fitting a (simplified) model of the microscope’s detection point spread function (PSF) to the image data, depth-dependent aberrations and variations of the PSF can compromise the localisation precision. One such detrimental aberration is caused by the refractive index mismatch between aqueous imaging buffer, cover glass and immersion medium, which occurs when standard immersion objectives and buffers are used. We have shown that we could suppress mismatch-induced aberrations by using glycerol-immersion objectives in conjunction with fused-silica cover glasses and imaging buffers with a high glycerol content, which ensures a uniform refractive index between sample and objective lens while still maintaining a comparatively high NA. This is especially beneficial for thick samples or schemes where fluorescent light is collected via two objectives facing each other, as these aberrations become more prominent deep in the sample. We have demonstrated the validity of this approach by successfully applying dual-objective dSTORM imaging to both microtubuli and nuclear pore complexes^7^.

The switching behaviour of fluorescent dyes in dSTORM, however, is highly dependent on the buffer composition. As our glycerol-immersion setup requires substantial amounts of glycerol in the buffer, its suitability for dSTORM has to be thoroughly ascertained. Therefore we present a quantitative analysis of dSTORM dyes to estimate the impact of glycerol on the switching properties.

## Results and discussion

### Alexa647 has excellent switching properties in glycerol-containing buffer

The dye Alexa Fluor 647, which is excited with red light at 647 nm, is widely used in dSTORM imaging due to its outstanding brightness. Together with 25 other dyes, its favourable switching properties were thoroughly investigated previously for aqueous buffer systems^4^. Four parameters were found to be decisive for good image quality: the photon number per switching event, the mean duty cycle, the survival fraction and the total number of switches^4^.

**Figure 1:**
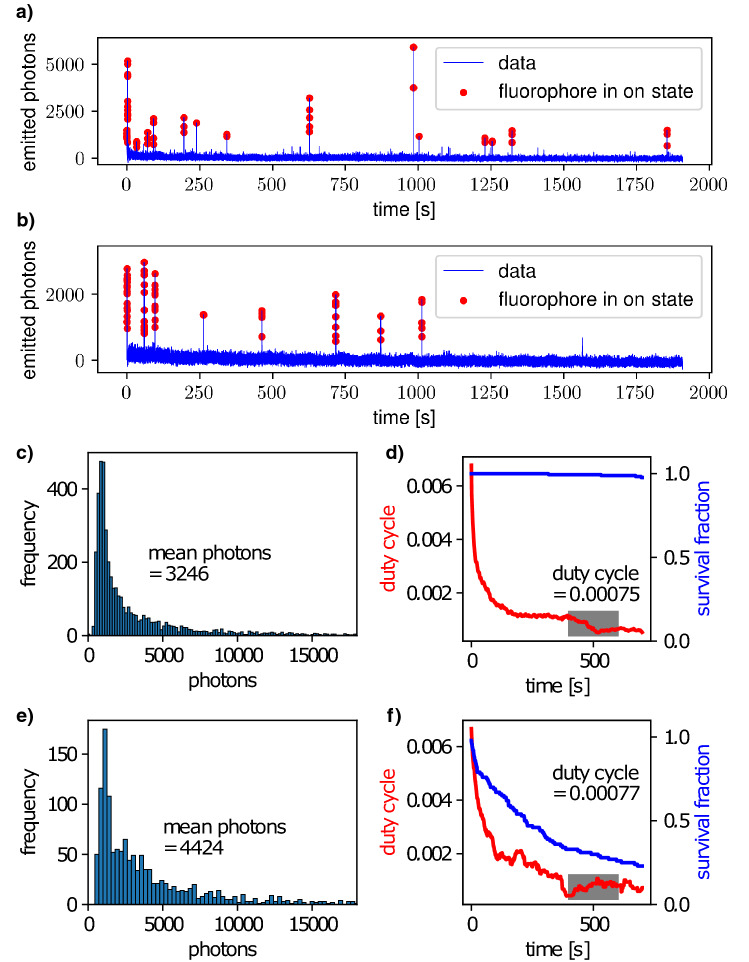
Evaluation of Alexa Fluor 647 measurements. (**a**) Exemplary fluorescent time trace for acquisition without activation laser, (**c**) photon distribution, (**d**) duty cycle and survival fraction. (**b**,**e**,**f**) The same for measurements with activation laser.

To measure these properties, we adhered single dye molecules to the cover glass and imaged them under dSTORM imaging conditions. Besides 10 mM MEA as reducing agent and an oxygen-scavenging system consisting of glucose, glucose oxidase and catalase, the imaging buffer contained about 55% glycerol to raise its refractive index to about 1.45. During measurement, the sample was continuously illuminated with 647 nm laser light at about $8.6\,\frac{\text {kW}}{\text {cm}^2}$ (see “Methods” section). Single photoswitching molecules were identified and their photon emission against time was investigated. As can be seen in Fig. 1a, most of the time the number of emitted photons is near zero, interrupted with short outbursts of high photon emission. The molecule is defined as fluorescing if the number of emitted photons per video frame is higher than ten times the standard deviation of the background fluctuations from single molecule time traces. Frames where a molecule is in the on state are indicated by red dots in the time traces in Fig. 1a,b.

The photon number per switch *N* is the average number of detected photons emitted in one such blinking event. For a well-suitable dye, it should be as high as possible as the localisation precision scales with $\frac{1}{\sqrt{N}}$^8,9^. The duty cycle $DC(t) = \frac{\sum _{i} \tau _{on, i}}{100\,\text {s}}$ is the fraction of time the molecule is on in a sliding 100 s interval, where the mean *DC* is calculated as the arithmetic mean between 400 s and 600 s. For good image quality, it should be low because a high *DC* increases the probability of observing not single but overlapping fluorescent molecules, degrading the fit precision. The survival fraction is the fraction of molecules from which fluorescence is still detected after 400 s. Here, a high survival fraction is desirable as it indicates good photostability of the dye. Lastly, the number of switches was defined as the total number in 2,000 s. A high number of switches is desirable as it allows to detect the same dye molecule multiple times and therefore to sample the stained structure repeatedly, resulting in improved measurement statistics.

In our glycerol-containing buffer, we measured on average $3{,}314 \pm 98$ photons per switch for Alexa Fluor 647 (Fig. 1c), a number that is comparable to aqueous buffers^4^. In our buffer, the duty cycle $DC = 0.00087 \pm 0.00017$ was slightly increased (Fig. 1d) compared to previous results, but still low enough to yield good image quality. With a survival fraction of $(96 \pm 4)$ % and $30 \pm 5$ switches, however, we obtained slightly improved values. In summary, we found that the switching properties of Alexa Fluor 647 were only slightly changed by glycerol addition, making it an excellent red-absorbing dSTORM dye in our proposed buffer system as well.

#### Influence of the activation laser

In dSTORM, the return rate of dye molecules from the non-fluorescent radical anion state to the fluorescent ground state is often increased by illumination with short-wavelength visible light^6^. Therefore, we also investigated the influence of activation laser light at 405 nm on the switching behaviour of Alexa Fluor 647 (see Fig. 1b and Table 1). Note that the first column of Table 1 provides information about the evaluation statistics: for instance, the first line (Alexa Fluor 647 (n.a.)) indicates that in total 233 fluorophores in 2 data stacks from 2 measurement days have been evaluated. If more than one measurement day was needed for gathering a sufficient amount of data, the samples have been prepared according to the same protocol as described in detail in “Methods” section.

**Table 1:** Switching properties of Alexa Fluor 647 with (a.) and without (n.a.) use of 405 nm activation laser.

Fluorophore	Evaluated fluorophores/number of measurements/time [days]	Photon number per switch	Equilibrium on-off duty cycle [$10^{-4}$]	Survival fraction after illumination for $400\,\text {s}$ [$\%$]	Mean number of switching cycles
Alexa Fluor 647 (n.a.)	233/2/2	$3{,}314\pm 98$	$8.7\pm 1.7$	$96\pm 4$	$30\pm 5$
Alexa Fluor 647 (a.)	531/5/2	$3{,}880\pm 190$	$6.0\pm 1.5$	$32\pm 15$	$13\pm 5$

We found that with $3{,}880 \pm 190$ the number of photons per switch was slightly higher (Fig. 1e) and with $DC = 0.00060 \pm 0.00015$ the duty cycle was somewhat improved as well, but the survival fraction drops to $(32 \pm 15)$ % (Fig. 1f) and the number of switches to $13 \pm 5$.

In comparable measurements with purely aqueous buffers, we measured in agreement with previous publications^4^ that the illumination with $405\,\text {nm}$ of Alexa Fluor 647 increases the *DC* in comparison to the non-activated case (see Supplementary Table 3). Furthermore, we could not observe a significant drop of the survival fraction in aqueous buffers so that the harsh drop described above is due to the glycerol. This indicates that for our setup, Alexa Fluor 647 should not be used with activation laser illumination as it leads to severe photobleaching.

#### Enzymatic activity in glycerol-containing buffer

As explained above, oxygen needs to be removed from the imaging buffer for dSTORM to ensure a sufficiently low return rate from the radical anion state to the fluorescent ground state. Furthermore, oxygen in the sample increases photobleaching because dye molecules in the triplet state can turn oxygen in the triplet (ground) state into reactive singlet oxygen, which then in turn destroys the fluorescent molecule^10^. Therefore, oxygen is removed from imaging buffers via catalase, glucose and glucose-oxidase. Glucose oxidase removes oxygen by catalysing its reaction with glucose to D-Glucono-1,5-lactone and hydrogen peroxide. In the next step, hydrogen peroxide is decomposed by catalase. Accordingly, it is vital for dSTORM imaging that the enzymes glucose-oxidase and catalase remain active in the buffer. But the high glycerol concentration which we employ might affect their correct folding^11^ or reduce enzymatic activity due to the high viscosity of glycerol^12^.

To check enzyme activity, we measured the switching properties of Alexa Fluor 647 in buffer without glucose-oxidase and catalase. The survival fraction was $(74 \pm 4)$ % and the number of switches $(18.9 \pm 0.8)$. Therefore, as expected, these properties were significantly lower without enzymes, while photon numbers and *DC* were not impaired. This shows that presence of these enzymes reduces photobleaching in spite of the high glycerol content.

### Switching properties of yellow-absorbing dyes

Among fluorophores excitable with light in the yellow colour range, to our knowledge there is no single dye that is unambiguously regarded as perfectly suited for dSTORM.

Therefore we analysed the switching behaviour of several different dyes to find out which work best in our measurement conditions. These dyes were excited with 561 nm laser light at about $7.3\,\frac{\text {kW}}{\text {cm}^2}$ (see “Methods” section).

#### Alexa Fluor 568 has best switching properties among yellow-absorbing dyes

**Figure 2:**
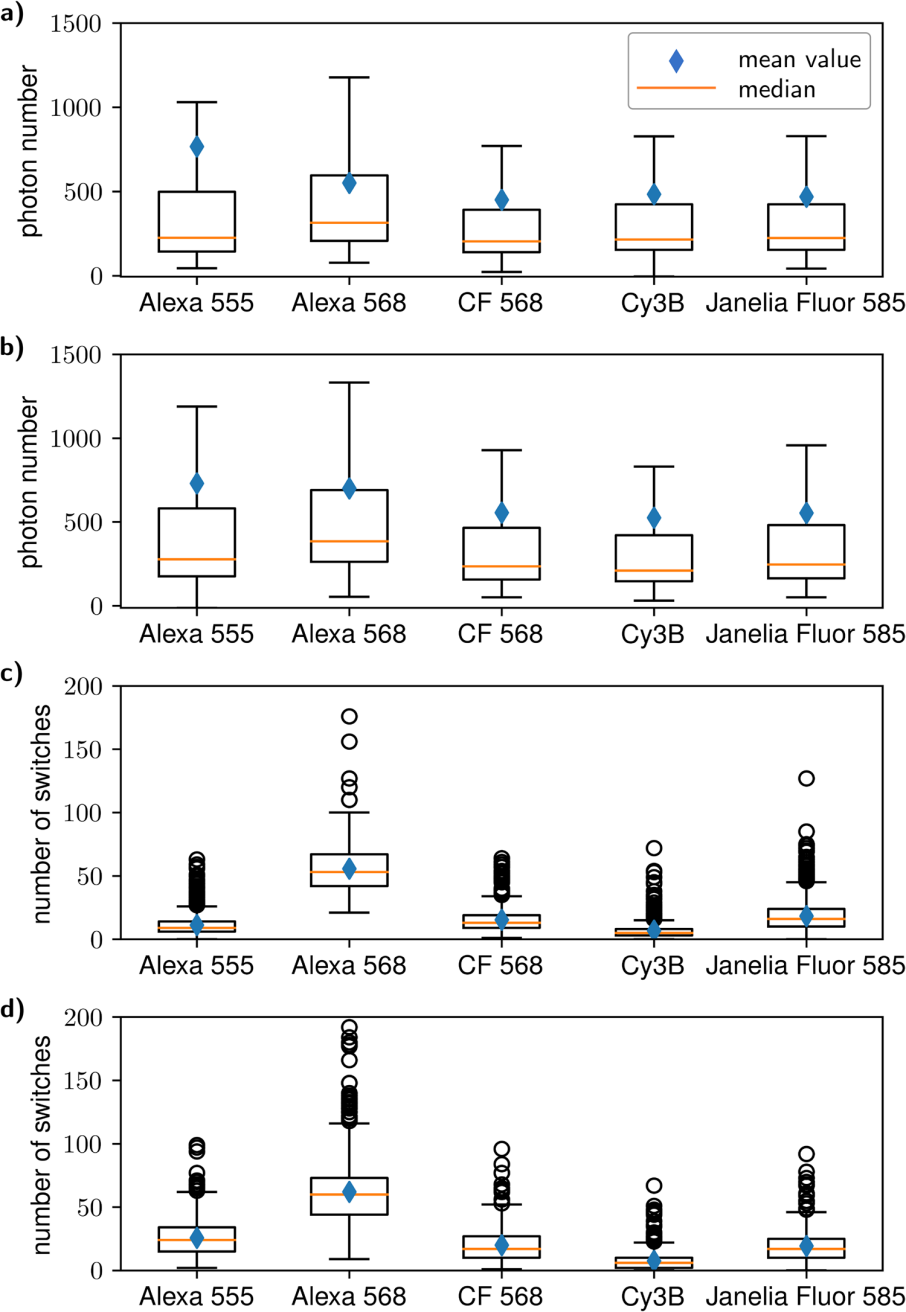
Boxplot representation of photon emission and number of switches for the characterised yellow-absorbing dyes. The boxes denote values from the lower quartile to the upper quartile whereas the whiskers extend over the whole data range with the restriction that they are not longer than 1.5 the boxlength. (**a**,**b**) Photon emission without and with 405 nm illumination, respectively. (**c**,**d**) The same for total number of switches.

We measured five dSTORM dyes which can be excited with yellow light of wavelength 561 nm: Alexa Fluor 555, Alexa Fluor 568, CF 568, Cy3B and Janelia Fluor 585 (see Fig. 2 and Supplementary Table 4). All of them showed photoswitching in glycerol-containing buffer, but emitted substantially less photons per switch than Alexa Fluor 647. The mean number of emitted photons is quite similar for all five dyes (about 500 photons), with Alexa Fluor 555 being the brightest with 765 ± 65 photons per switch without activation laser. But as Alexa Fluor 568 features an outstandingly high number of switching cycles (with ∼ 60 cycles, it switches several times more than the other dyes), it performs best in our imaging conditions. It also displays the highest survival fraction and median photon number per switch. Its drawback is its comparatively high *DC* (0.00126 ± 0.00007 without and 0.0016 ± 0.0005 with activation laser) but it should still be low enough to yield good image quality. Contrary to Alexa Fluor 647, in our hands this dye performs better with activation laser.

#### Switching properties of Alexa Fluor 568 in conventional imaging buffer

Unlike Alexa Fluor 647, Alexa Fluor 568 emitted significantly less photons per switch in our measurements than reported in literature for aqueous buffers^4^. With $552 \pm 19$ photons, we only observed 20% of the photon number that was reported before. To identify the cause for this discrepancy, we measured the switching properties of Alexa Fluor 568 in conventional aqueous buffer with exactly the same composition as in the study mentioned above. We measured $922 \pm 37$ photons per switch, a duty cycle $DC = 0.0005 \pm 0.0001$, a survival fraction of $(88 \pm 2)$ % and on average $24 \pm 3$ switching cycles. This means that in our conditions, the photon number is indeed reduced to about 60%, but this disadvantage is compensated by an improved survival fraction and number of switching cycles.

Since the addition of glycerol does not have such a large effect, the low number of photons in our experiments must have other causes as well. One reason for the differences might be the slightly deviating method to calculate the photon numbers (see “Methods” section). Additionally, we used different fluorescence filter sets. In particular, in front of our cameras we employ notch filters which filter out the light of all lasers installed in the setup and are not optimised especially for the use with Alexa Fluor 568 (see Supplementary Fig. 1). Due to this, the detected intensity of Alexa Fluor 568 should be lower by about one third in our setup. The combined effect of all these factors may explain the discrepancy between previous results and our own findings.

### A sealed sample geometry improves survival fractions

**Figure 3:**
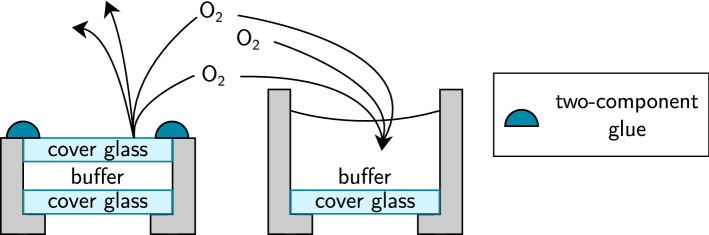
Explanatory model for high survival fraction of Alexa Fluor 568 and 647. In normal sample geometry, buffer is pipetted onto a single cover glass so that there is a permanent surface between buffer and ambient air. This enables that reactive oxygen diffuses into the buffer which initially has got a low oxygen concentration. In contrast, the dSTORM sample at the left is sealed with a second cover glass and two-component glue so that oxygen does not easily diffuse into the buffer.

As described in the previous sections, with our setup we measure very high survival fractions of almost 100% during the 400 s illumination period for Alexa Fluor 647 and 568, meaning that there is almost no photobleaching. These values are higher than reported before^4^. CF 568 and Janelia Fluor 585 also barely photobleach. Our measurement without oxygen scavenger system indicates that the high survival fractions are likely due to a low oxygen content in the sample. This is also consistent with the fact that reactions with oxygen lead to phototoxic singlet oxygen and hydrogen peroxide. The reason why the oxygen content in our samples could be unusually low might be the sample geometry. For use with our dual-objective microscope, we employ sealed samples where the imaging buffer is covered with a second cover glass and the gap between the two cover glasses is tightly closed with two-component glue (Fig. 3, left panel). Therefore oxygen cannot easily diffuse into the buffer and the oxygen concentration should remain low for a long time after oxygen is removed by the oxygen scavenging system. In contrast to this, dSTORM is often performed in open chambers where the imaging buffer is exposed to ambient air (Fig. 3, right panel) so that oxygen can diffuse into the buffer.

To test this hypothesis, we analysed the switching properties of Alexa 647 in such an open well geometry. This experiment had to be performed on a different setup because in the dual-objective dSTORM microscope, samples are inserted vertically which is not possible for open chambers. For the open well geometry, we used cover slips with a diameter of $18\,\text {mm}$ supplied with $465\,\upmu \text {l}$ of buffer (for buffer composition, cf. “Methods” section below). The buffer has been prepared directly before the measurements so that there were less than $10\,\text {min}$ between the end of buffer preparation and the beginning of the measurements.

Compared to this, the sealed sample geometry requires only $16\,\upmu \text {l}$ of buffer and more time is needed between adding the buffer and starting the experiment due to sealing the sample, drying of the two-component glue and alignment with two objectives. We do not observe a difference in the results even after waiting several hours between sample preparation and starting the experiment.

For the open well geometry, we obtained on average a duty cycle $DC = 0.00043 \pm 0.00002$, a survival fraction of $(27 \pm 2)$ % and a number of switching cycles of $12.6 \pm 1.9$. These values are drastically lower than in the case of sealed samples, indicating considerably higher photobleaching.

### Conclusion

We could show that glycerol addition to the imaging buffer does not markedly deteriorate the switching properties of Alexa Fluor 647 and 568. This means that this dye pair is suitable for dSTORM with glycerol immersion objectives and the accompanying glycerol-containing buffer, thus allowing to make use of the advantages of refractive index matching this system possesses.

Interestingly, we measured very low photobleaching for these dyes, probably due to our use of sealed samples which reduce oxygen influx into the buffer. But for other dyes (e.g. Cy3B), the survival fraction was reduced in our measurements, which shows that besides the sample geometry there are other factors which influence photobleaching. Although our approach is not able to quantify the impact of these other sources on photobleaching, our findings point out that the sample geometry proposed here may become a new possibility to improve dSTORM image quality.

## Methods

### Camera calibration

Due to ageing processes, the EMCCD camera gain can degrade over time. For this reason, the EM gain defined in the camera software may no longer agree with the true, physical EM gain and it becomes necessary to calibrate the camera anew to exactly convert camera counts into detected photons. For this task, fluorescent spheres, 40 nm dark red carboxylate-modified Fluo Spheres (F8789, Invitrogen Ltd., Paisley, UK) for the red colour channel and 100 nm Tetra Speck microspheres (T7279, Invitrogen Ltd., Paisley, UK) for other channels, have been imaged with different illumination powers in order to measure the whole dynamic range between background fluctuations and saturation. Thus, several image stacks of 100 frames have been acquired and the pixel mean values $n_{ic}$ and variances $\sigma _{n_{ic}}^2$ have been calculated for determining the gain *g* from

$$\begin{aligned} \sigma _{n_{ic}}^2=\frac{2g}{f}n_{ic}, \end{aligned}$$

where f is an analogue-to-digital factor^13^. The gains that have been determined for the two EMCCDs from the slope of a variance-mean-diagram are $(23.9\pm 0.5)$, $(31.7\pm 0.6)$ when the input gain value is 40, and $(69.3\pm 1.4)$, $(92.1\pm 1.8)$ when the input gain is 100. Hence, camera calibration is necessary to avoid systematic errors in the calculation of photon numbers.

### Sample preparation

Single fluorescent molecules were prepared by firstly gluing a $30\,\text {mm}$ diameter fused silica cover glass (Leica Microsystems, Wetzlar, Germany) into a metal ring (Feinmechanische Werkstatt im Physikalischen Institut, Münster, Germany). Together with a second silica cover glass of the same size, the sample was treated in a plasma cleaner as described before^7^. After this step, $50\,\upmu \text {l}$ poly-l-lysine (P4707, SIGMA ALDRICH CHEMIE GmbH, Steinheim, Germany) were pipetted onto the sample and left on the cover glass for 10 minutes incubation time. The remaining poly-l-lysine was sucked from the cover glass and then, the sample was rinsed with deionised water. Next, $50\,\upmu \text {l}$ of fluorescent dyes diluted in $100\,\text {mM}$ sodium bicarbonate (pH 8.3) were added onto the sample and again left for 10 minutes. The fluorophores have been chosen as NHS esters so that they covalently bind to the primary amine functional groups offered by the poly-l-lysine. This bond is sufficiently strong to avoid hydrolysis^14^. Finally, after carefully rinsing with deionised water again, $50\,\upmu \text {l}$ of a diluted solution of fluorescent beads were added to the sample and left for 1 min incubation. The exact dilutions of fluorescent dyes in $100\,\text {mM}$ sodium bicarbonate and of beads in deionised water had to be adjusted for each dye and are documented in the Supplementary Table 1.

For dSTORM conditions, samples were embedded in imaging buffer based on a buffer containing 200 mM 1,4-piperazinediethanesulfonic acid (A1079, AppliChem GmbH, Darmstadt, Germany), supplied with 40% (w/v) glucose (6887.1, Carl Roth GmbH + Co. KG, Karlsruhe, Germany) and adjusted to a pH of 7.2 using NaOH. Glycerol (G5515-100ML, SIGMA ALDRICH CHEMIE GmbH, Steinheim, Germany) was added to the buffer until its refractive index was in the range of 1.45. The glycerol concentration then was about 55% of volume. The refractive index was measured with a refractometer (DR201-95, A. Krüss Optronic, Hamburg, Germany). Directly before applying the buffer to the sample, it was supplied with 0.8$\frac{mg}{ml}$ glucose oxidase (G2133-10KU, SIGMA ALDRICH CHEMIE GmbH, Steinheim, Germany), 0.08$\frac{mg}{ml}$ catalase (C1345-1G, SIGMA ALDRICH CHEMIE GmbH, Steinheim, Germany) and 10 mM MEA (also known as cysteamine, 30070-10G, SIGMA ALDRICH CHEMIE GmbH, Steinheim, Germany).

For imaging, $16\,\upmu \text {l}$ of dSTORM imaging buffer were added to the samples and they were mounted with a second cover glass onto a custom metal sample holder.

### Data acquisition

Data acquisition was carried out on our custom modular dSTORM microscope with two objectives described previously^7^. In short, four lasers (LuxX 647-140, 647 nm, 140 mW, Omicron Laserage Laserprodukte GmbH, Rodgau, Germany; Cobolt Jive, 561 nm, 100mW, Cobolt AB, Solna, Sweden; Cobolt Calypso, 491 nm, 100mW, Cobolt AB, Solna, Sweden; PhoxX 405-60, 405 nm, 60mW, Omicron Laserage Laserprodukte GmbH, Rodgau, Germany) served to excite and photoactivate fluorophores. The resulting light intensities in the focal plane for the three excitation lasers were about $8.6\,\frac{\text {kW}}{\text {cm}^2}$, $7.3\,\frac{\text {kW}}{\text {cm}^2}$ and $4.8\,\frac{\text {kW}}{\text {cm}^2}$, respectively. When the 405 nm activation laser was used, its incident intensity was increased

automatically via an acousto-optical tunable filter, where the transmitted intensity *T* was augmented according to

1 $$\begin{aligned} T = T_{i} + \left( T_{e} - T_{i}\right) \cdot \frac{\text {e}^{\,p \frac{t}{t_a}} - 1}{\text {e}^{\,p} - 1} \end{aligned}$$

where *t* is the time since starting the acquisition, $t_a$ the total acquisition time, $T_{i} = 2$%, $T_{e} = 15$% and $p = 2.5$. In this manner, the intensity in the focal plane increased from about $0.9\,\frac{\text {W}}{\text {cm}^2}$ to $24\,\frac{\text {W}}{\text {cm}^2}$.

Fluorescent light was collected via two glycerol immersion objectives (HCX PL APO 100×/1.35 GLYC CORR, Leica Microsystems, Wetzlar, Germany) which are used in conjunction with fused silica cover glasses and a glycerol-water mixture adjusted to a refractive index of about 1.45 as immersion medium.

The microscope is equipped with two different detection pathways. In the experiments presented here, we used the astigmatic detection pathway where the light captured by each objective is imaged onto an electron-multiplying charge-coupled device (iXon3 897, Andor Technology Ltd., Belfast, UK) and cylindrical lenses in the detection beam path enable 3D imaging. Although 3D detection was not necessary for the experiments presented here, the cylindrical lenses were left in the detection beam path to facilitate finding the focal plane. Notch filters are installed in front of the cameras to remove laser light from the detection path (QuadLine Rejectionband ZET405/488/561/640, AHF analysentechnik AG, Tübingen, Germany) and additional emission filters (red-absorbing dyes: 700/75 ET Bandpass, yellow-absorbing dyes: 617/73 BrightLine HC, 525/50 ET Bandpass, both from AHF analysentechnik AG, Tübingen, Germany) permit to choose the spectral range of the detected fluorescence.

The measurement of switching properties in an open sample geometry was conducted on an inverted fluorescence microscope customised for dSTORM described elsewhere^15^. For this setup, we employed an oil-immersion objective (CFI Apo TIRF 100XC Oil) and normal N-BK7 cover glasses (41001118, Assistent Karl Hecht GmbH & Co., Sondheim, Germany). For every measurement, 60000 frames with an acquisition time of 30 ms were acquired.

### Analysis of switching properties

To calculate the switching properties, we closely followed the framework of a study which analysed the switching behaviour of 26 dyes in aqueous buffers^4^. The only notable difference concerned the number of photons per switching cycle where we applied an alternative calculation both due to our own measurement conditions and due to theoretical concerns.

Firstly, we acquire double the data amount as two objectives and two EMCCDs are used for data acquisition. Since we adjust our system such that nearly the same image is falling on both detectors, we determine an estimate for the photon number per switch for each camera. We then form an arithmetic mean of the two determined values to calculate the photon histograms and mean values presented in this article.

Secondly, the aforementioned publication suggests to create histograms of the photon number per switch and to obtain the mean number of photons from fitting a decaying exponential function to these histograms. Theoretical expectations according to boson statistics suggest, however, that the detection of the emitted photon number has to be represented by an asymmetric probability density function (pdf). This also becomes visible in the data when photon histograms are displayed with a finer binning than chosen in the reference study. An exponential fit may be suitable to account for the slowly decaying edge on the right hand side of the pdf, but not for the quickly falling edge on the left hand side (cf. Fig. 1). For this reason, we chose to calculate the photon number from the arithmetic mean of our measurements.

From the raw data image stacks, signals have been identified in the first few hundred images with our previously described particle localisation algorithm^7^. Spatial drift during the experiments was measured by localising sub-resolution sized fluorescent beads added to the samples. This time-dependent drift was subtracted from the particle coordinates. Any identified signal lying within five pixels ($160\,\text {nm}$ per pixel) of another signal was omitted according to the reference study^4^. By integrating over a $7\times 7$ pixel area, fluorescent time traces have been calculated from which all other parameters can be derived. For each time trace, fluorophores have been defined to be switched on when their signal exceeded the background fluctuations plus 10 times the standard deviation. The time-dependent duty cycle was calculated as $DC(t) = \sum _i \tau _{on,i}/100\,\text {s}$ where the time window covered the interval $[t,t+100\,\text {s}$]. Molecules identified as bleached have been excluded in the calculation of *DC*(*t*). The scalar values for the *DC* as given in Fig. 1 were calculated as arithmetic mean from all *DC*(*t*) lying between $400\,\text {s}$ and $600\,\text {s}$. The photon mean numbers and the equilibrium *DC* printed in the figures deviate from those given in tables like Table 1 because in the first case, the images are evaluations of a single measurement whereas the tables show mean values over all measurements of the corresponding fluorescent dye.

The time-dependent survival fraction was calculated as the number of molecules that are not yet bleached divided by the total number of detected molecules. A single molecule was defined to be bleached after its final on-switch. The mean number of switching cycles was determined from a time window of $2{,}000\,\text {s}$ as an arithmetic mean over all detected molecules.

## Supplementary information

Supplementary Information 1.

## Data Availability

The data sets generated and analysed during the current study are available from the corresponding authors on reasonable request.
