# Supplementary material for: Switching behaviour of dSTORM dyes in glycerol-containing buffer
Source: Sci Rep. 2020 Aug 13;10:13746. doi: 10.1038/s41598-020-70335-0 (PMC7426933; doi:10.1038/s41598-020-70335-0)
Supplement: Supplementary file 1 — Supplementary Information 1. [file 41598_2020_70335_MOESM1_ESM.pdf]

Switching behaviour of dSTORM dyes in glycerol-containing buffer

Nora C. Goossen-Schmidt[1,2,+]**, Marco Schnieder**[1,2,+]**, Jana Hüve**[1,2,*]**, and Jürgen Klingauf**[1,2,*]

[1]Fluorescence Microscopy Facility Münster (FM)[2], Institute of Medical Physics and Biophysics, Center For NanoTechnology (CeNTech), Heisenbergstraße 11, 48149 Münster, Germany
[2]Department of Cellular Biophysics, Institute of Medical Physics and Biophysics, University of Münster, Robert-Koch-Straße 31, 48149 Münster, Germany
[*]jana.hueve@uni-muenster.de, klingauf@uni-muenster.de
[+]these authors contributed equally to this work

Supplementary Information

Details about Sample Preparation

The dilutions defined in Supplementary Table 1 have been applied according to the protocol in the Methods section for the preparation of single fluorescent molecules bound to poly-L-lysine on the cover slip surface. More detailed information about the fluorescent dyes and beads used for the experiments are summarised in Supplementary Table 2.

Supplementary Table 1. Quantities for preparation of a sample with single fluorescent dyes.

fluorophore	stock solution in DMSO [mg/ml]	dilution with 100 mM sodium bicarbonate	beads	dilution in deionised water
Alexa Fluor 555	10.0	$1:10^7$	580/605	$5:10^7$
Alexa Fluor 568	10.0	$1:10^8$	580/605	$5:10^7$
Alexa Fluor 647	10.0	$5:10^7$	660/680	$5:10^7$
CF 568	≈ 10.0 ($1\,\mu$mol/$100\,\mu$l)	$1:10^8$	580/605	$5:10^7$
Cy3B	1.0	$1:10^5$	580/605	$5:10^7$
Janelia Fluor 585	3.3 (2 mg/$600\,\mu$l)	$1:10^7$	580/605	$5:10^7$

Supplementary Table 2. Fluorescent dyes and beads that have been used for sample preparation.

designation in text	product information
Alexa Fluor 555	Alexa Fluor^{TM} 555 NHS ester, 1 mg, A37571, Thermo Fisher Scientific Inc., Waltham, USA
Alexa Fluor 568	Alexa Fluor^{TM} 568 carboxylic acid succinimidyl ester, 1 mg, A20003, Thermo Fisher Scientific Inc., Waltham, USA
Alexa Fluor 647	Alexa Fluor^{TM} 647 NHS ester, $3 \cdot 100\,\mu$g, A37573, Thermo Fisher Scientific Inc., Waltham, USA
CF 568	CF^{R} 568 succinimidyl ester, $1\,\mu$mol, 92131, Biotium Inc., Fremont, USA
Cy3B	Cy^{TM}3B NHS mono-reactive dye, 1 mg, PA63101, GE Healthcare, Buckinghamshire, UK
Janelia Fluor 585	Janelia Fluor^{R} 585, SE, 2 mg, 6418, Tocris Bioscience, Bristol, UK
beads 580/605	Fluo Spheres^{TM} carboxylate-modified $0.04\,\mu$m, red (580/605), F8793, Invitrogen Ltd., Paisley, UK
beads 660/680	Fluo Spheres^{TM} carboxylate-modified $0.04\,\mu$m, dark red (660/680), F8789, Invitrogen Ltd., Paisley, UK

Influence of the activation laser

We found that in our glycerol-containing-buffer, illumination with an activation laser reduced the survival fraction and duty cycle of Alexa Fluor 647. As this result differed from a previous study with aqueous buffer[4], we measured these parameters again for a buffer that contained no glycerol. All other measurement conditions were identical. Without glycerol, we did not observe the dramatic drop of the survival fraction and the duty cycle was increased, not reduced. This shows that for some dyes, glycerol addition to the buffer indeed requires to adapt the measurement protocol.

Supplementary Table 3. Duty cycle and survival fraction for Alexa Fluor 647 with (a.) and without (n.a.) use of 405 nm activation laser for buffer without any glycerol addition.

fluorophore	evaluated fluorophores/ number of measurements	equilibrium on-off duty cycle [10^{-4}]	survival fraction after illumination for 400 s [%]
Alexa Fluor 647 (n.a.)	288/2	2.2 ± 0.1	97.9 ± 0.5
Alexa Fluor 647(a.)	463/3	3.6 ± 0.1	97 ± 3

Switching properties of yellow-absorbing dyes

Supplementary Table 4 shows the photon number per switching event, the mean duty cycle, the survival fraction and the total number of switches for all dyes in the yellow-absorbing range which were investigated in this study.

Supplementary Table 4. Switching properties of Alexa Fluor 555, Alexa Fluor 568, CF 568, Cy3B and Janelia Fluor 585 with (a.) and without (n.a.) use of 405 nm activation laser.

fluorophore	evaluated fluorophores/ number of measurements/ time [days]	photon number per switch	equilibrium on-off duty cycle [10^{-4}]	survival fraction after illumination for 400 s [%]	mean number of switching cycles
Alexa Fluor 555 (n.a.)	708/4/1	765 ± 65	3.0 ± 0.3	83 ± 3	11.9 ± 0.3
Alexa Fluor 555 (a.)	633/4/1	705 ± 85	8.0 ± 0.7	92.6 ± 1.3	27 ± 2
Alexa Fluor 568 (n.a.)	332/3/2	552 ± 19	12.6 ± 0.7	99.7 ± 0.3	58.6 ± 1.6
Alexa Fluor 568 (a.)	692/5/1	703 ± 15	16 ± 5	99.86 ± 0.14	65 ± 8
CF 568 (n.a.)	586/3/1	452 ± 32	4.7 ± 0.4	94 ± 2	16.2 ± 0.4
CF 568 (a.)	557/3/1	556 ± 36	6.1 ± 0.2	95.7 ± 0.8	21.0 ± 0.1
Cy3B (n.a.)	645/5/2	484 ± 25	2.3 ± 0.2	82 ± 2	7.3 ± 0.4
Cy3B (a.)	649/5/2	518 ± 42	2.3 ± 0.4	76 ± 2	8.0 ± 0.4
Janelia Fluor 585 (n.a.)	1279/7/2	474 ± 13	4.3 ± 0.5	97.4 ± 0.5	19 ± 2
Janelia Fluor 585 (a.)	580/3/1	551 ± 28	4.1 ± 0.7	96.4 ± 0.5	20.3 ± 0.9

Dependence of photon numbers on measurement setup

While the equilibrium on-off duty cycle, the survival fraction and the mean number of switching cycles only depend on a detection of signals and recognizing fluorophores as being switched on, the calculated photon number per switch depends on the intensity of the detected signal and thus on the individual microscope.

With respect to the yellow-absorbing dyes, the photon numbers are comparably low as compared to other studies[4]. Besides the difference in the calculation method of the mean photon number per switch (cf. Methods section of the main article), there is also an impact of our filters on the brightness of the detected signal. For the detection of yellow-absorbing molecules, we use a notch filter (QuadLine Rejectionband ZET405/488/561/640), AHF analysentechnik AG, Tübingen, Germany) and an additional orange filter (617/73 BrightLine HC, AHF analysentechnik AG, Tübingen, Germany). Supplementary Figure 1 illustrates for the example of Alexa Fluor 568 that the compromise of using the notch filter to filter out four different laser wavelengths has the effect that only 54.7 % of the spectrum emitted by Alexa Fluor 568 is transmitted to the detector. This impact always has to be considered if photon numbers from different setups and sample preparations shall be compared to each other.

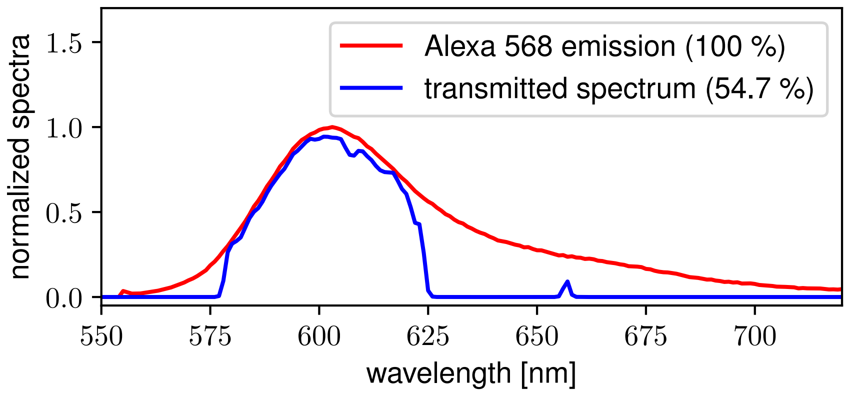

Supplementary Figure 1. Comparison between Alexa Fluor 568's emission spectrum (red) and the product of this spectrum and the filter transmission functions (blue).

Overview of Fluorescent Dye Analysis

For the sake of better comparability, mainly mean values and medians are used in the main article to refer to our results. In the Supplementary Figures 2 to 6, overviews about the fluorescent dye evaluations are given for getting more detailed information. In the above mentioned figures, exemplary fluorescent time traces, photon histograms over all measurements as well as the time dependent *DC* and the time dependent survival fraction are shown.

In the time traces, single data points that are identified as an on-switched molecule are marked with red dots. As the duration of a fluorescent blinking event is less than about 100 ms, the peaks are visible as a straight line in the chosen illustration although they are extended in time. For example, the rightmost peak in Figure Supplementary 2 a) is marked with three red dots meaning that the peak is identified as an on-switch being extended over three time steps, i.e. $\approx$ 100 ms.

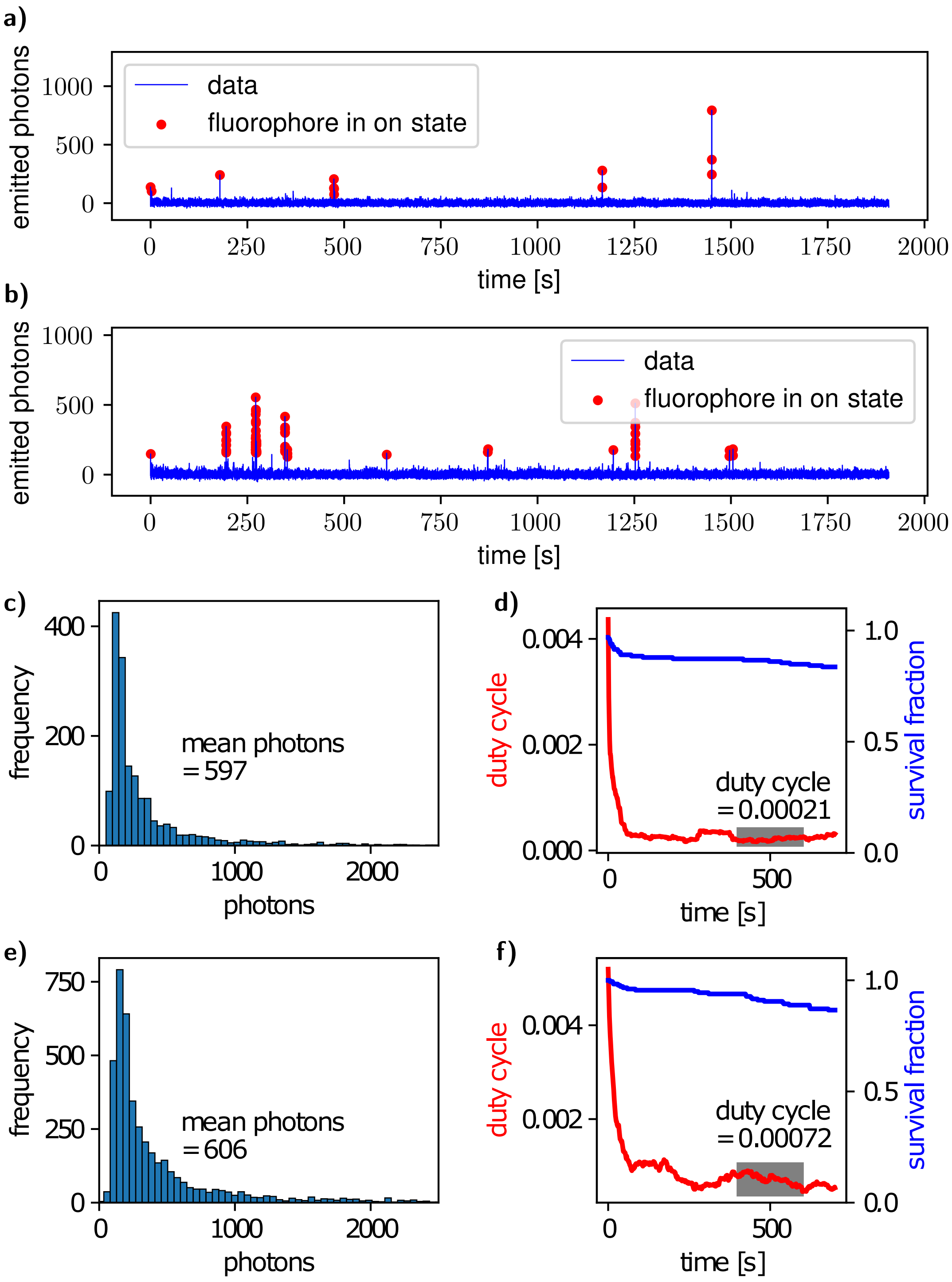

Supplementary Figure 2. Evaluation of **Alexa Fluor 555** measurements. a) Exemplary fluorescent time trace for acquisition without activation laser, c) photon distribution, d) duty cycle and survival fraction. b),e),f): the same for measurements with activation laser.

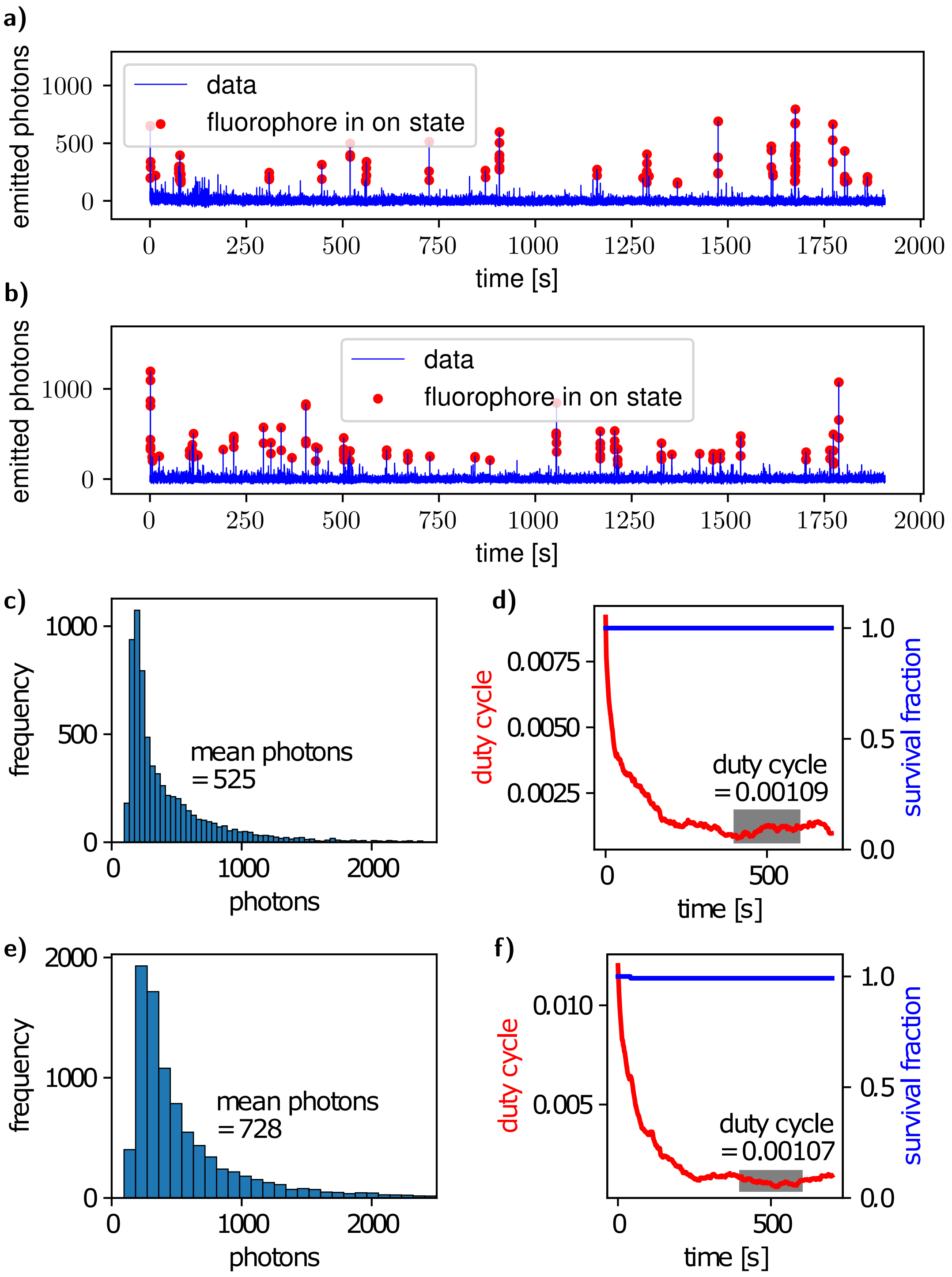

Supplementary Figure 3. Evaluation of **Alexa Fluor 568** measurements. a) Exemplary fluorescent time trace for acquisition without activation laser, c) photon distribution, d) duty cycle and survival fraction. b),e),f): the same for measurements with activation laser.

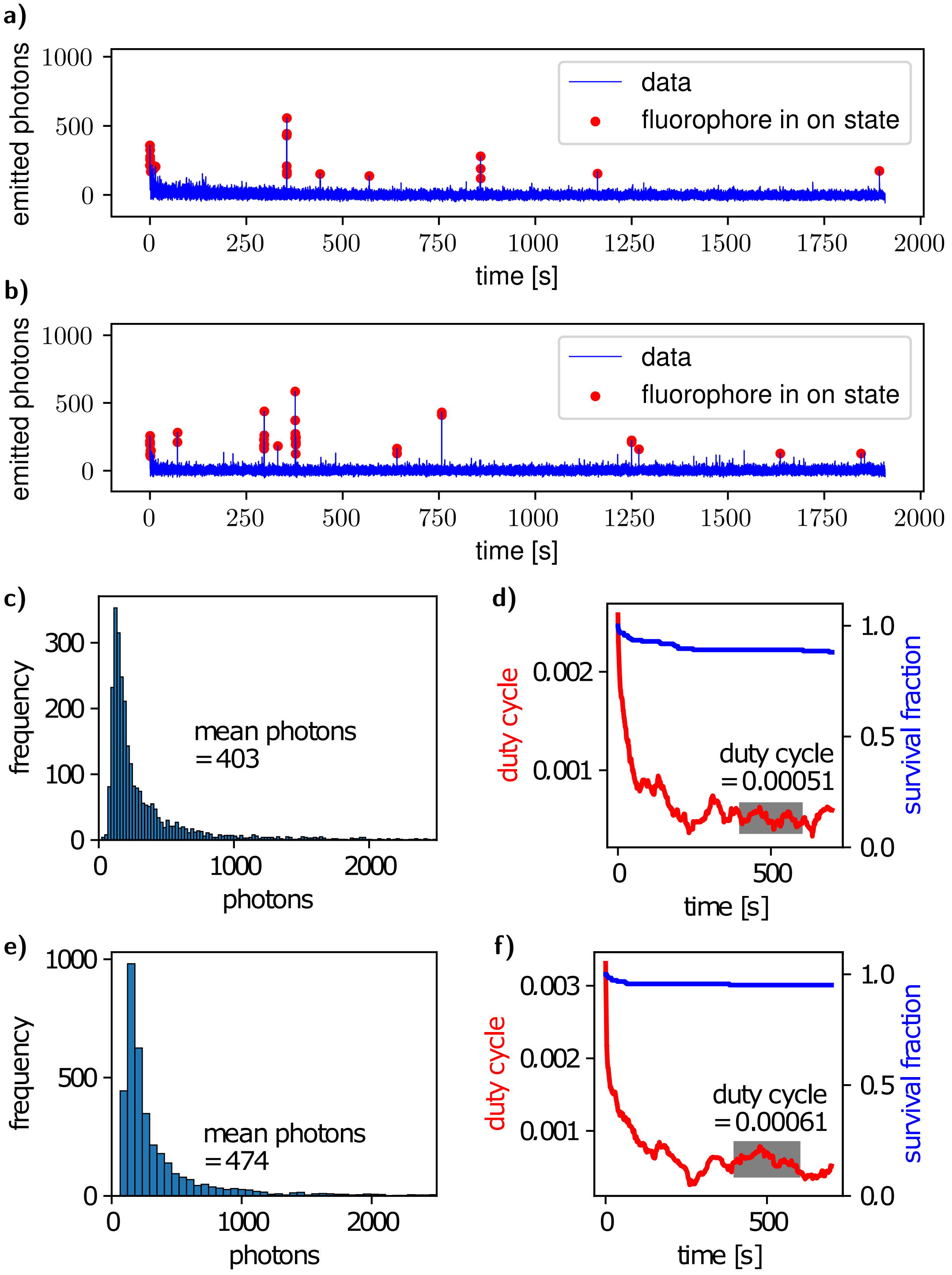

Supplementary Figure 4. Evaluation of **CF 568** measurements. a) Exemplary fluorescent time trace for acquisition without activation laser, c) photon distribution, d) duty cycle and survival fraction. b),e),f): the same for measurements with activation laser.

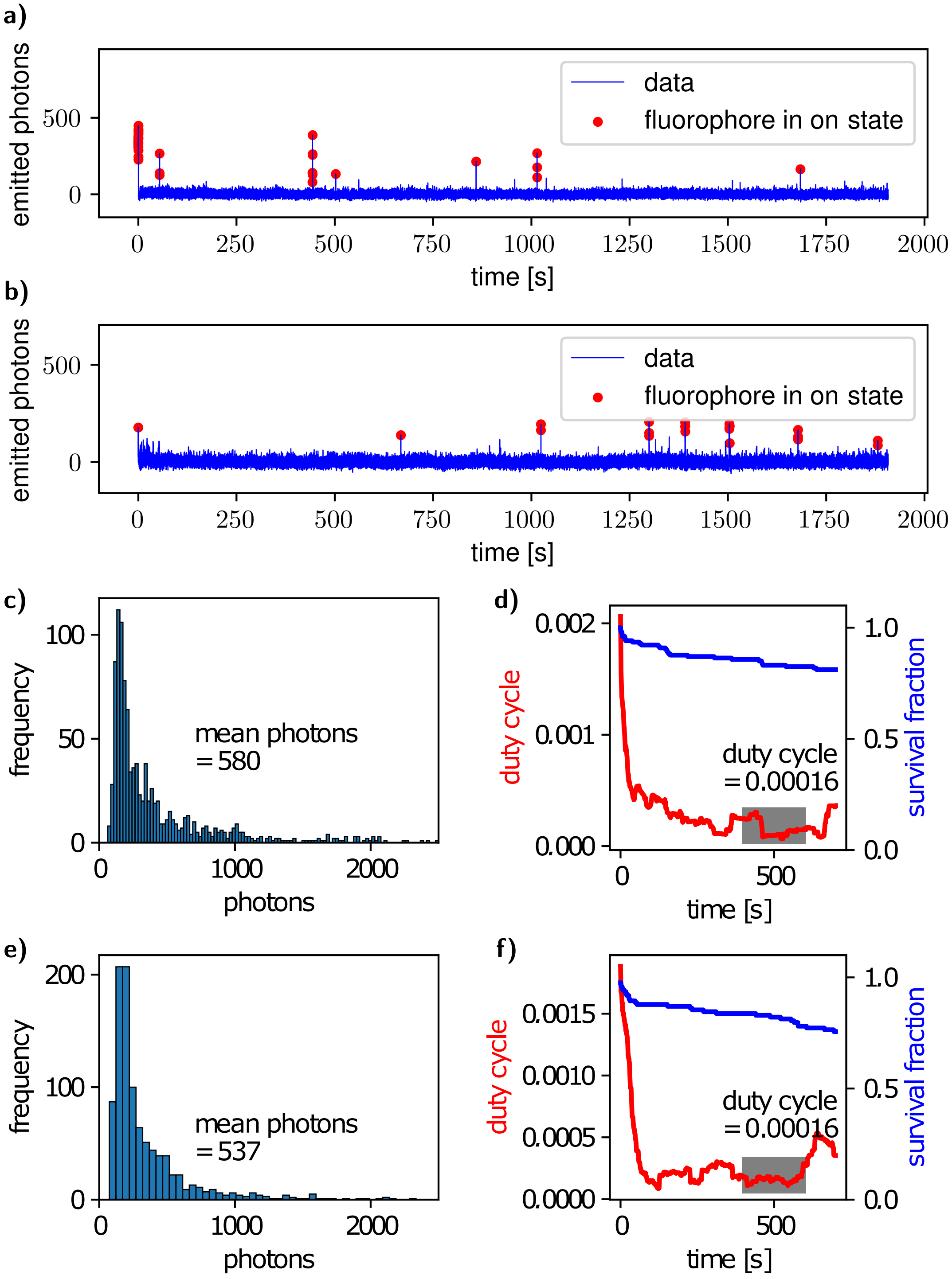

Supplementary Figure 5. Evaluation of **Cy3B** measurements. a) Exemplary fluorescent time trace for acquisition without activation laser, c) photon distribution, d) duty cycle and survival fraction. b),e),f): the same for measurements with activation laser.

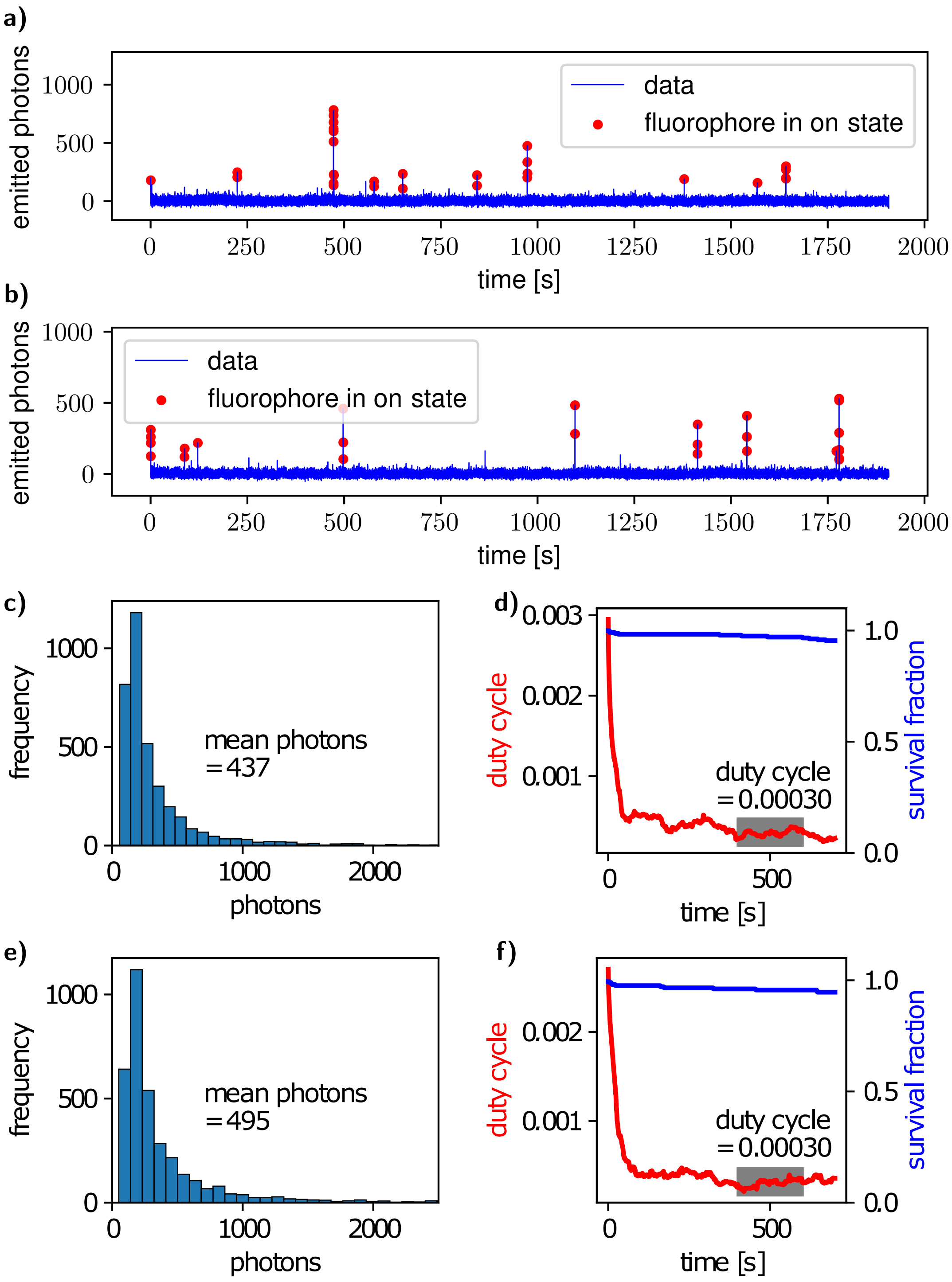

Supplementary Figure 6. Evaluation of **Janelia Fluor 585** measurements. a) Exemplary fluorescent time trace for acquisition without activation laser, c) photon distribution, d) duty cycle and survival fraction. b),e),f): the same for measurements with activation laser.
